# Design and Validation of a Short Novel Estradiol Aptamer and Exploration of Its Application in Sensor Technology

**DOI:** 10.3390/molecules29020535

**Published:** 2024-01-22

**Authors:** Hongyan Jin, Yan Cheng, Fanli Kong, He Huang, Zhenjun Yang, Xinyi Wang, Xinxia Cai, Jinping Luo, Tao Ming

**Affiliations:** 1 Obstetrics and Gynecology Department, Peking University First Hospital, Beijing 100034, China; 2 State Key Laboratory of Transducer Technology, Aerospace Information Research Institute, Chinese Academy of Sciences, Beijing 100190, Chinajpluo@mail.ie.ac.cn (J.L.); 3 University of Chinese Academy of Sciences, Beijing 100049, China; 4 State Key Laboratory of Natural & Biomimetic Drugs, School of Pharmaceutical Sciences, Peking University, No. 38 Xueyuan Road, Haidian District, Beijing 100191, China; 5 Institute of Transplantation Medicine, School of Medicine, Nankai University, Tianjin 300190, China

**Keywords:** aptamer, 17β-estradiol, microscale thermophoresis, colorimetric, electrochemical

## Abstract

The specific and sensitive detection of 17β-estradiol (E2) is critical for diagnosing and treating numerous diseases, and aptamers have emerged as promising recognition probes for developing detection platforms. However, traditional long-sequence E2 aptamers have demonstrated limited clinical performance due to redundant structures that can affect their stability and recognition ability. There is thus an urgent need to further optimize the structure of the aptamer to build an effective detection platform for E2. In this work, we have designed a novel short aptamer that retains the key binding structure of traditional aptamers to E2 while eliminating the redundant structures. The proposed aptamer was evaluated for its binding properties using microscale thermophoresis, a gold nanoparticle-based colorimetric method, and electrochemical assays. Our results demonstrate that the proposed aptamer has excellent specific recognition ability for E2 and a high affinity with a dissociation constant of 92 nM. Moreover, the aptamer shows great potential as a recognition probe for constructing a highly specific and sensitive clinical estradiol detection platform. The aptamer-based electrochemical sensor enabled the detection of E2 with a linear range between 5 pg mL^–1^ and 10 ng mL^–1^ (R^2^ = 0.973), and the detection capability of a definite low concentration level was 5 pg mL^–1^ (S/N = 3). Overall, this novel aptamer holds great promise as a valuable tool for future studies on the role of E2 in various physiological and pathological processes and for developing sensitive and specific diagnostic assays for E2 detection in clinical applications.

## 1. Introduction

17β-estradiol (E2) is a crucial hormone for maintaining female physiological functions, including secondary sexual characteristics, pregnancy, and bone metabolism [1,2]. The measurement of serum E2 is essential for diagnosing many diseases, such as delayed sexual development or precocious puberty, abnormal menstrual cycles, menopause, ovulation induction, infertility, ectopic pregnancy, and gynecomastia [3,4].

Currently, immunoassays and mass spectrometry (MS) are the most common laboratory tests for detecting E2 [5,6,7,8]. The immunoassay is based on the specific binding between antibodies and antigens [9,10]. Estrone (E1) and estriol (E3) are the metabolites and structural analogs of E2, interfering with the determination of E2 in the immunoassay [11,12]. In addition, when the sample concentration is higher than 1000 pg/mL, diluting the sample before the assay is often necessary. This dilution process may also affect the accuracy of the results [13]. Moreover, the potential interference from E2 isomers has still not been effectively resolved. MS has also been used for E2 determination due to its high sensitivity, high selectivity, and wide dynamic range [14,15,16]. However, these methods are more complicated, expensive, and time-consuming than conventional approaches. Both immunoassays and MS are not suitable for the need for quick and instant clinical tests. Developing a high-sensitivity and specificity biosensor for detecting E2 is necessary for efficient clinical diagnosis. Electrochemical sensing stands out for its high sensitivity, rapid response time, and selectivity in detecting target molecules at low concentrations [17]. The technology allows for miniaturization and portability, enabling the development of affordable, handheld devices for on-site testing and point-of-care diagnostics. Its compatibility with nanomaterials enhances performance, and the sensors find applications across diverse fields, including environmental monitoring, clinical diagnostics, and food safety. Electrochemical sensors offer real-time monitoring capabilities, ease of operation, and environmental friendliness, making them versatile and effective tools for a range of analytical needs [18].

Nucleic acid aptamers, selected by the systematic evolution of ligands via the exponential enrichment technique, are oligonucleotides with high affinity and specificity for target molecules, including proteins, toxins, antibiotics, drugs, and heavy metals [19,20,21]. Aptamers have been developed as recognition elements in biosensors due to their conformational changes in response to the target molecule [22,23,24]. In 2007, Kim’s group developed the first 76-mer E2 nucleic acid aptamer via SELEX and constructed an electrochemical biosensor by immobilizing the aptamer on a gold electrode with a minimum detection limit of 0.01 nM [25]. This aptamer has been used to construct a variety of analyses in E2 detection [26,27]. In our previous work, we constructed a 76-mer paper-based universal microfluidic electrochemical platform, which can achieve a detection limit of 10 pg/mL E2 under optimized experimental conditions [14]. It also achieved a linear fitting curve from 10 pg/mL to 4800 pg/mL in clinical serum samples. However, the redundant structure in the 76-mer long sequence affects its stability and interference with the target recognition [28,29,30,31]. At the same time, the longer sequence also increases the synthetic cost. Chen’s group scissored the 76-mer aptamer and screened for the E2 binding motifs [31]. Two shorted aptamers, E9 and E10, were reported effective in E2 recognition. Based on the molecular dynamics calculations of the E2 aptamer, we speculated that base T in E9 and E10 may be involved in E2 interactions [32].

In this paper, we reconstructed the E2 aptamer to enhance its specific binding ability with E2. Microscale thermophoresis (MST) analysis was used to evaluate the interaction strength between the aptamer and E2. After that, we validated the affinity of the modified aptamer for E2 using a colorimetric assay, which allows for the easy observation of the result with the naked eye. Furthermore, we constructed an unlabeled electrochemical aptamer-based biosensor (EAB) with the validated aptamer, which has a simple structure. The sensor was evaluated for E2 detection in blood samples using electrochemical assays. Our results demonstrate that the proposed aptamer has excellent specific recognition ability and great potential as a recognition probe for constructing a highly specific and sensitive clinical estradiol detection platform.

## 2. Results and Discussions

### 2.1. Novel Aptamers for E2 and Its Affinity

In the Mfold structure of the 76-mer aptamer, four stem-loop motifs are suggested, from which two loops were found to be responsible for the specific binding to E2 by Chen’s group, as shown in two short aptamers E09 and E10, respectively. In our research, the stem was first examined with new sequences in HEV1 and HEV2, respectively. Secondly, two dangling sequences were added to the stem-loop to adapt biosensors’ construction in different evaluation approaches. The two-dimensional structural simulation diagrams of HEV1 and HEV2 are shown in Figure S2A and Figure S2B, respectively.

**Figure 1:**
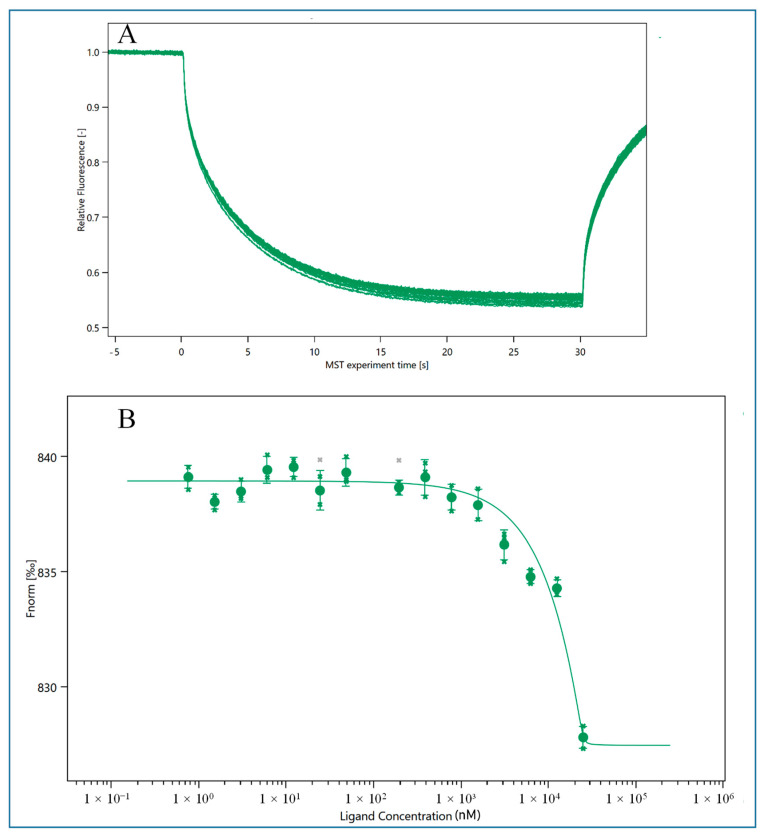
MST results of HEV1 binding with E2. (**A**) MST detection result of HEV1; (**B**) MST binding curve of HEV1.

As shown in Figure 1, the MST binding curve of HEV1 is plotted [33]. When HEV1 reaches a certain concentration of 500 nM, the fluorescence value of the sample will show a rapid decrease after reaching the plateau period, which indicates that HEV1 can bind to E2, and the dissociation constant (K_D_ value) is 92.76 ± 66.02 nM as calculated by the calculation function that comes with the system. The K_D_ value of the M70 was 98 ± 56 nM, indicating that the truncated HEV1 had a similar binding ability to the parental aptamer M70. The comparison of K_D_ value between kinds of aptamers is listed in Table S1. And in this experiment, the aptamer HEV2 cannot bind with E2. Therefore, in the following experiments, we will select HEV1 as the recognition probe for E2 to develop sensors.

### 2.2. Colorimetric Assay

#### 2.2.1. Chemical Characterization of AuNPs

Colorimetry is a visual and sensitive detection method. Here, we can intuitively understand the binding ability of HEV1 to E2 through the colorimetry colorimetric assay. The schematic diagram of the colorimetric method is shown in Figure S3.

The AuNPs were characterized using transmission electron microscopy (TEM) and UV–visible absorption spectrum. As shown in Figure S4A, the nanogold particle size of the nanogold particles is relatively homogeneous, and the distribution is relatively uniform. As shown in Figure S4B, the diameter of the AuNPs is about 15 nm.

#### 2.2.2. Optimization of NaCl Concentration

**Figure 2:**
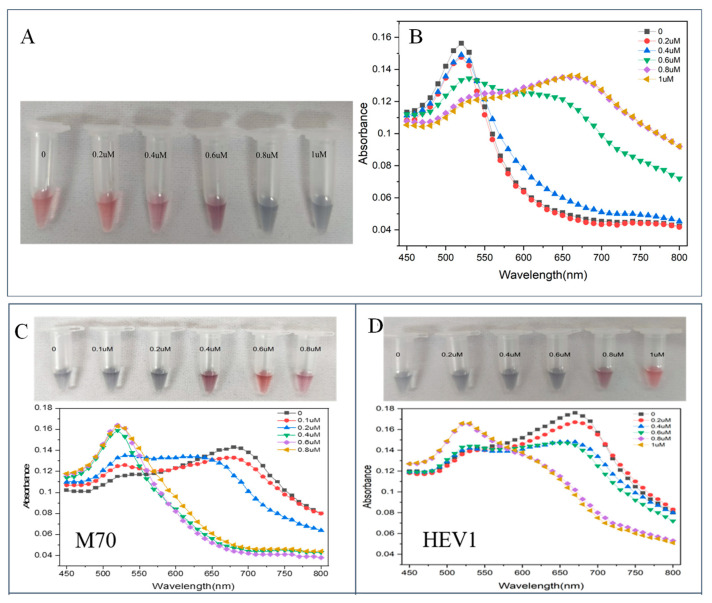
(**A**) Visual observation results of different C_NaCl_; (**B**) absorption spectra of different C_NaCl_. (**C**) color development and detection spectra after adding different concentrations of M70; (**D**) color development and detection spectra after adding different concentrations of HEV1.

The NaCl concentration (C_NaCl_) affects the dispersion of AuNPs and the sensitivity of the sensor. As C_NaCl_ increases, the absorption peak of AuNPs decreases at 520 nm, and the peak absorption at 660 nm increases. In the optimization experiment (Figure 2A,B), 10 μL of different C_NaCl_ were added to a mixture of 50 μL of ultrapure water and 50 μL of AuNPs. After incubation for 5 min at room temperature, the color change of the AuNPs solution was observed and detected by spectrophotometry, and the absorbance values of the solution at 520 nm and 660 nm were recorded. The minimum C_NaCl_ that can completely induce the aggregation of AuNPs was determined to be 0.8 μM.

#### 2.2.3. Optimization for Aptamer Concentration

Aptamers protect AuNPs from aggregation. In the presence of NaCl, the low concentration of aptamer (C_Apt_) did not provide sufficient protection to AuNPs, resulting in the aggregation of AuNPs and the solution turning blue. As C_Apt_ increased, the protective effect of the aptamer on AuNPs was enhanced, which prevented AuNPs from aggregating in the NaCl solution and changed the solution color from blue to red. The optimization experiments for C_Apt_ are shown in Figure 2C,D. At the optimized C_NaCl_, 25 μL of different C_Apt_ were added to the system containing 25 μL of ultrapure water and 50 μL of AuNPs and mixed uniformly with a pipette. After 10 min of incubation, 10 μL of the optimal C_NaCl_ was added and mixed. After incubation for 5 min at room temperature, the color change of the AuNPs solution was observed and detected using spectrophotometry, and the absorbance values of the solution at 520 nm and 660 nm were recorded. The C_Apt_ that can prevent the system from turning blue was determined.

#### 2.2.4. Analytical Performance of the Colorimetric Sensor

**Figure 3:**
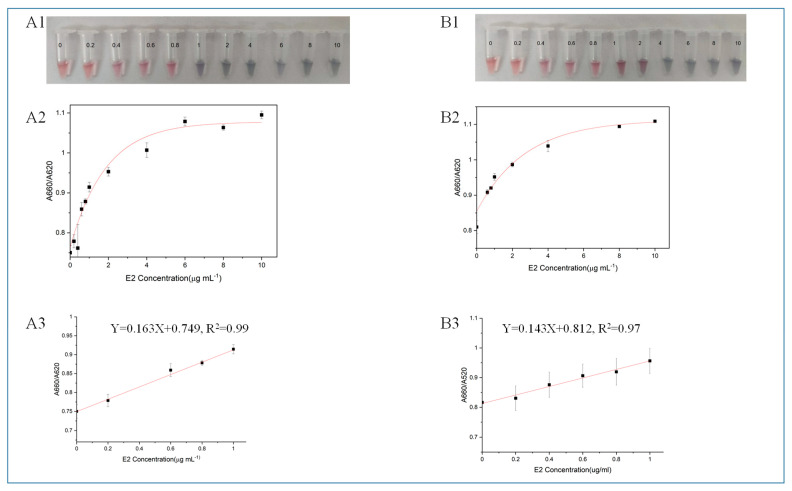
(**A**) Detecting E2 with M70. (**A1**) The actual detection diagram, (**A2**) the relationship diagram between the absorbance ratio A660/A520 of the system and C_E2_ and (**A3**) the linear regression curve. (**B**) Detecting E2 with HEV1. (**B1**) The actual detection diagram, (**B2**) the relationship diagram between the absorbance ratio A660/A520 of the system and C_E2_ and (**B3**) the linear regression curve.

According to the mechanism of AuNPs-based colorimetric aptamer sensors, the aggregation of AuNPs causes the solution color to change from red to purple or blue. As shown in Figure 3, as the concentration of E2 (C_E2_) increases, the absorbance decreases at 520 nm and the absorbance value at 660 nm increases.

The ratio of A660/A520 was calculated as the basis for quantitative analysis. When using HEV1 for colorimetric detection, there is a good linear relationship between C_E2_ and A660/A520 ratio when the C_E2_ is 0.2 to 1 μg/mL (y = 0.143x + 0.812, R^2^ = 0.97). E2-free samples were analyzed, and it was determined that the detection capability of a definite low concentration level using HEV1 colorimetry was 0.109 μg/mL. When using the M70 for colorimetric inspection, the detection capability of a definite low concentration level is 0.151 μg/mL. The comparison shows that HEV1 is more sensitive than M70 when detecting E2 using the colorimetric method.

### 2.3. Electrochemical Assay

#### 2.3.1. Characterization of Synthesized rGO/THI/AuNP Nanocomposites

**Figure 4:**
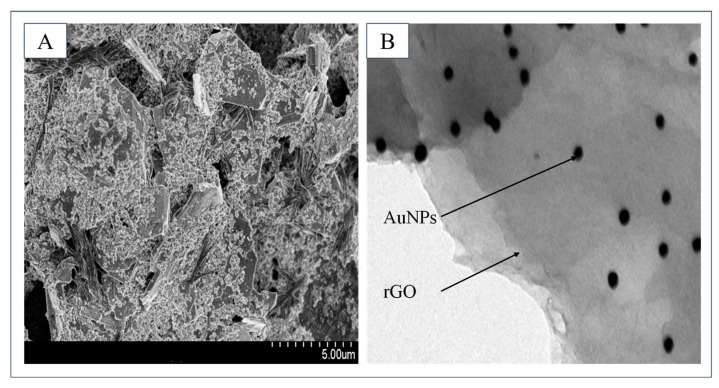
(**A**) The SEM result of the modified electrode surface. (**B**) The TEM result of the synthesized nanocomposites.

To characterize the synthesized nanocomposites, the nanocomposites modified electrode and the synthesized nanocomposites are characterized via scanning electron microscope (SEM) and transmission electron microscopy (TEM). As shown in Figure 4A, after modification, the electrode surface presents a rough and uneven structure, which effectively increases the specific surface area of the electrode. As shown in Figure 4B, the synthesized nanocomposites are clearly observed. AuNPs are uniformly distributed on rGO without any aggregation phenomenon.

#### 2.3.2. Electrochemical Performance of the Aptasensor

**Figure 5:**
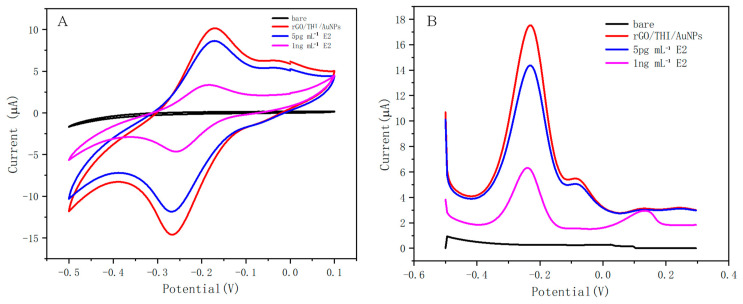
(**A**) CV response of working electrodes subjected to different treatments. (**B**) DPV response of working electrodes subjected to different treatments.

Firstly, we needed to examine whether the electrochemical response of the working electrode was improved after modification with nanomaterials. In this experiment, the bare electrode, the working electrode modified with nanocomposite, the modified electrode after incubation with 5 pg/mL E2, and the modified electrode after incubation with 1 ng/mL E2 were characterized by CV and DPV, respectively, and the results are shown in Figure 5.

The CV response is shown in Figure 5A. The bare electrode did not exhibit oxidation or reduction behavior when the nanomaterials were not modified, while after modification with the nanomaterials, the working electrode exhibited a good electrochemical response, and a clear redox peak could be observed, which was generated due to the electrochemical activity of thionine. When different C_E2_ were added, the peak current intensity decreased with increasing C_E2_, which indicates that the electron transfer from the dielectric to the working electrode was affected by the binding of E2 to the aptamer. This is consistent with our assay principle. The results of CV indicate that the fabrication of this electrochemical sensor was successful.

The DPV response is shown in Figure 5B. The results obtained from the DPV study were correlated with the CV performed under similar conditions. The working electrode with modified nanomaterials exhibited a significant DPV response with a peak current of 17.52 μA compared to the bare working electrode. The magnitude of the DPV response gradually decreased with an increasing concentration of the added E2. The results of the DPV test indicate that the fabrication of this electrochemical sensor was successful.

#### 2.3.3. Analytical Performance of the Aptasensor

**Figure 6:**
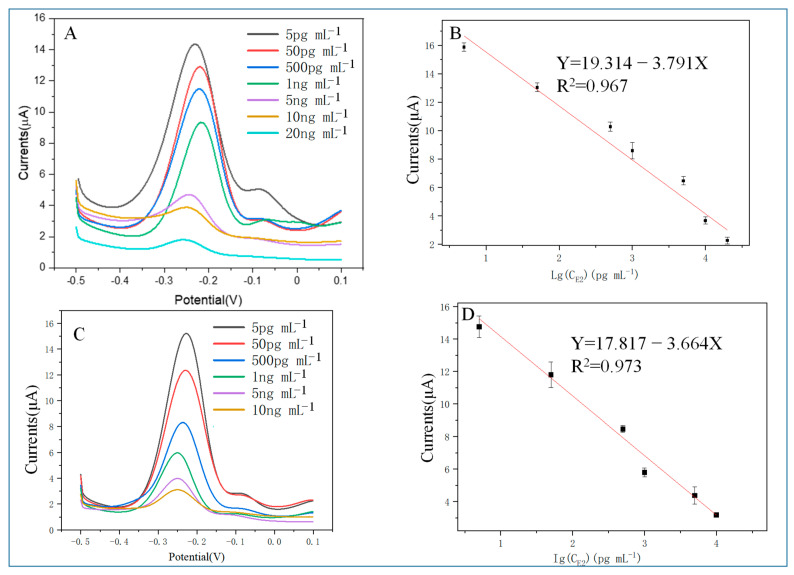
(**A**) The DPV response of the M70 sensor when detecting different C_E2_; (**B**) M70 sensor detection result: calibration curve between DPV response and logarithmic C_E2_; (**C**) DPV response when HEV1 sensor detects different C_E2_; (**D**) HEV1 sensor detection result: calibration curve between DPV response and logarithmic C_E2_.

The analytical performance of the aptasensors fabricated through M70 and HEV1 is shown in Figure 6. When C_E2_ increases, the peak current gradually decreases, and a linear correlation is found.

In the concentration range of 5 pg/mL to 20 ng/mL, the I_DPV_ (μA) = 19.314 − 3.791 × LgC_17β-E2_ (pg/mL) (R^2^ = 0.967) for the M70 sensor with a detection capability of a definite low concentration level of 5 pg mL^−1^ (S/N = 3), and the linear relationship for the HEV1 sensor was I_DPV_ (μA) = 17.752 − 3.643 × LgC_17β-E2_ (pg/mL) (R^2^ = 0.973), with a detection capability of a definite low concentration level of 5 pg mL^−1^ (S/N = 3). The HEV1 short chain aptamer performs similarly to that of M70 with E2 standard solution.

#### 2.3.4. Repeatability and Selectivity of the HEV1-Based Aptasensor

The DPV response to paper chips that have been incubated with 1 ng/mL of E2 was measured to assess the reproducibility of this sensor. As shown in Figure S6A, the peak currents of these three paper chips were 5.54 μA, 5.58 μA, and 5.59 μA, respectively, with a coefficient of variation of 0.475%, indicating that the HEV1 aptamer sensor has good reproducibility.

As shown in Figure S6B, the variation of DPV response of the sensor was within 5.12% when mixed with other high-concentration structural analogs (E1, E3, and progestin) for detection, indicating that the HEV1 aptamer sensor has good selectivity.

#### 2.3.5. Analytical Results of Serum Samples

The practicality and analytical reliability of the paper-based aptasensor were further investigated by assaying different serum samples spiked with different C_E2_.

Figure S5 shows the calibration plots of peak current (I_DPV_) and C_E2_ in clinical serum samples for the detection of E2 by the M70 aptasensor and HEV1 aptasensor, respectively.

HEV1 sensor: I_DPV_ (μA) = 5.553 − 1.083 × LgC_E2_ (pg mL^−1^) (R^2^ = 0.973).

M70 sensor: I_DPV_ (μA) = 7.096 − 1.542 × LgC_E2_ (pg mL^−1^) (R^2^ = 0.981).

Therefore, it can be concluded that the HEV1 aptamer performs similarly to the M70 aptamer when detecting standard solutions of E2 in serum.

As shown in Table 1, the reference concentrations were obtained from a hospital chemiluminescent immunoassay analyzer, and the detection results of HEV1 based aptasensor were similar to reference concentrations for different clinical blood samples, with relative errors of −17.69% to 9.61%, indicating the accuracy of the sensor in detecting clinical serum samples.

## 3. Materials and Methods

### 3.1. Reagents and Apparatus

Hydrogen tetrachloroaurate (III) tetrahydrate (HAuCl_4_·4H_2_O) and trisodium citrate dihydrate (Na_3_C_6_H_5_O_7_) were purchased from Sinopharm Chemical Reagent Co., Ltd. (Shanghai, China). Dimethylsulfoxide (DMSO) was obtained from Nanjing Chemical Reagent Co., Ltd. (Nanjing, China). Reduced graphene oxide (rGO) was provided by XFNANO (Nanjing, China), and thionine (THI) was obtained from Bailingwei company (Beijing, China). The 17β-E2 was obtained from Abcam (Cambridge, UK). Phosphate-buffered saline (PBS) and 2-mercaptohexano (MCH) were obtained from Sigma-Aldrich Corp. (St. Louis, MO, USA). All the reagents were of analytical reagent grade and used as received. The clinical serum samples were approved by the Medical Ethics Committee of Peking University First Hospital. Aptamers were constructed using Mfold Web Server [34] and synthesized by Sangon Biological Engineering Technology & Co. Ltd. (Shanghai, China).

All experiments, including cyclic voltammetry (CV), differential pulse voltammetry (DPV), and electrochemical AC impedance (EIS), were conducted on an Autolab electrochemical workstation (Herisau, Switzerland). The binding affinity study was investigated on a Miniature Thermoelectrophoresis Apparatus NT.115 (Nano Temper Technologies (GmbH, Munich, Germany). Ultrapure water was obtained from a Michem Ultrapure Water apparatus (Michem, Chengdu, China, resistivity > 18 MΩ). A Xerox ColorQube 8570 digital wax printer (Xerox, Norwalk, CT, USA) was adopted to create microfluidic channels. The electrode surface morphology was observed by an S-3500 Scanning Electron Microscope (S-3500, Hitachi, Tokyo, Japan). Other apparatus, including an ultrasonic generator (KH2200E, Hechuang, China), an transmission electron microscope (Tecnai G2 F30 S-TWIN, FEI, Sunnyvale, CA, USA), an atomic force microscope (Dimension Icon, Bruker AXS, Karlsruhe, Germany), a UV-1700 UV–vis Spectrophotometer (Shimadzu, Kyoto, Japan), and a PHS-3C Precision pH Meter (Leica Devices Factory of Shanghai, Shanghai, China) were also adopted.

### 3.2. Modification for E2 Aptamer

**Figure 7:**
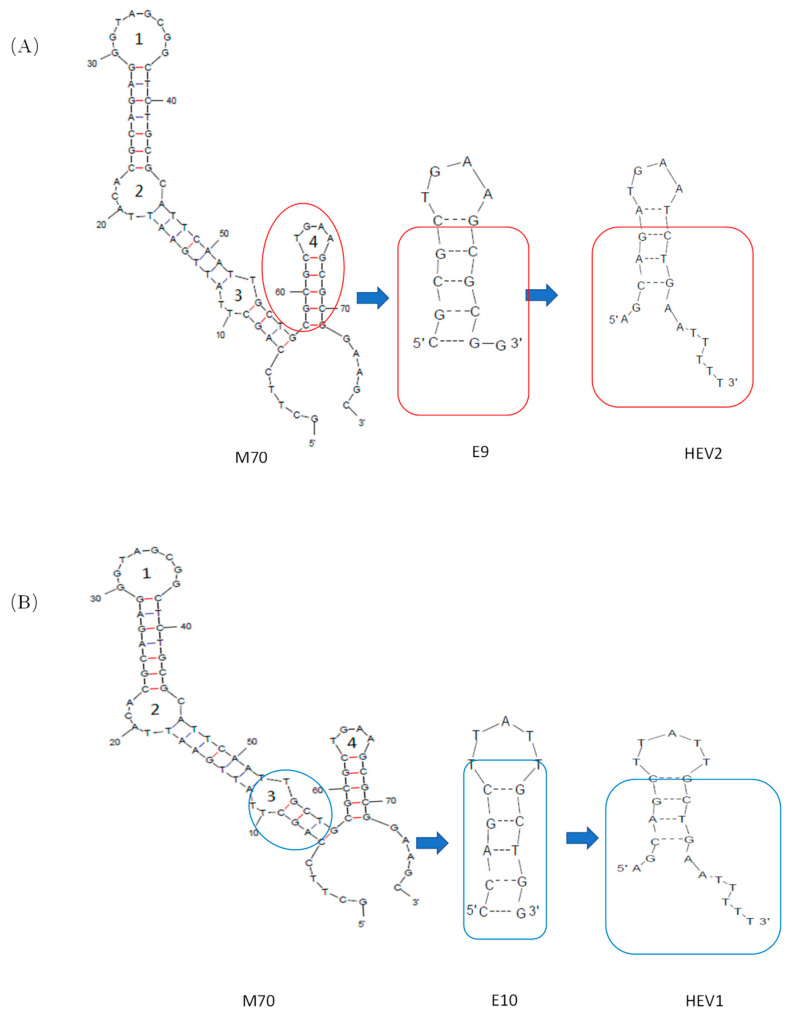
The schematic of short-aptamer modification of E2. (**A**) Scheme for Cutting M70 Adapters to Obtain HEV2; (**B**) Scheme for Cutting M70 Adapters to Obtain HEV1.

A new E2 aptamer was designed based on the secondary structure of E2 aptamer, namely the E10 (5′-CCAGCTTATTGCTGG-3′), as reported by Chen et al. [31]. As shown in Figure 7, the secondary structures of the modified aptamer were obtained using the Mfold Web Server. Based on the molecular dynamics calculations of E2 aptamer, we speculate that base T in E9 and E10 may be involved in E2 interactions, including the aromatic π between E_2_’s benzene ring and heterocyclic bases van der Waals force interaction between π interaction and other functional groups. Thus, as shown in Figure 1, the double helix composition of the stem was modified to keep the base composition of the ring domain unchanged for E9 and E10 and named aptamers HEV2 and HEV1, respectively. A 76-mer aptamer (5′-GCTTCCAGCTTATTGAAT TACACGCAGAGGGTAGCGGCTCTGCGCATTCAATTGCTGCGCGCTGAAGCGCGGAAGC-3′) from Kim’s group, named M70 in this study, was examined as an internal reference.

### 3.3. Preparation of MST Experiments for Novel Aptamer

#### 3.3.1. Preparation of the Reagents

Microscale thermophoresis (MST) is a powerful technique used to analyze biomolecular interactions. By leveraging the thermophoretic effect, MST employs an infrared laser to locally heat molecules, inducing their directional movement. The ensuing measurements can be analyzed using MO Affinity Analysis software (MO.AffinityAnalysis_x86_2.3.0.7385), which facilitates the automatic calculation of the KD value. The procedure of the MST is as follows:

The optimal volume for a single binding reaction is 20 μL, consisting of 10 μL of aptamer working fluid and 10 μL of respective estrogen diluents, facilitating convenient pipetting and minimizing experimental errors. Aptamer buffer is prepared by mixing 20 mM Tris (pH 7.6), 300 mM NaCl, 5 mM MgCl_2_, and 0.01% Tween 20. The aptamer working solution is created by dissolving the novel aptamer following reagent instructions and diluting it to a working concentration of 500 nM for subsequent testing. For the estrogen test solution, estrogen is dissolved in anhydrous ethanol, diluted to 2000 nM using ddH_2_O, and then mixed with aptamer buffer in sixteen labeled centrifuge tubes. Specifically, 20 μL of E2 (2000 nM) is placed in tube 1, and 10 μL of aptamer buffer is added to tubes 2 through 16. The liquid in tube No. 1 is transferred to tube No. 2, mixed by repeated pipetting, and then 10 μL of the mixed solution from tube No. 2 is transferred to tube No. 3. This process continues until it is diluted to tube No. 16, with each tube retaining a total of 10 μL of the test solution. Subsequently, 10 μL of the adapter working solution is added to each of the 16 tubes containing the estrogen test solution, thoroughly mixed, and the test samples are incubated with the added adapter for 30 min. Finally, the 16 hydrophilic capillary glass tubes are vertically inserted into the 16 test samples, and the capillary tube-containing tray is placed into a Monolith NT.115Pico instrument.

#### 3.3.2. Capillary Scanning and Detection

In preparation for the formal MST measurement, it is imperative to conduct a preliminary scan of the capillary glass tube. This step serves to detect fluorescence molecules adhering to the capillary wall, enabling the identification of potential issues such as pipetting errors or fluorescence quenching effects. Consequently, pre-capillary scanning expedites the identification of non-standard operations and facilitates the optimization of buffer solutions and sample concentrations.

Upon initiating the Nano Temple Control software (NT.115), configure the LED channel for cy5-dyne to “red”, setting the LED power to 20%. Commence the capillary scanning process. Following this, distribute capillaries with varying concentrations of estrogen to their respective tubes.

For the first capillary, designate the maximum estrogen concentration (resulting in a final concentration of 1000 nM). Adjust the MST power to 80% for optimal signal strength. Allow the instrument to execute fluorescence detection for 5 s using default settings, followed by a 30 s MST recording. Subsequently, record fluorescence for an additional 5 s to monitor the reverse diffusion of molecules.

### 3.4. Fabrication of AuNPs-Based Colorimetric Sensor for the Detection of E2

#### 3.4.1. Preparation of the AuNPs

Monodisperse, quasi-spherical AuNPs with sizes of 15 nm were prepared via a citrate reduction strategy [35]. Typically, 1 mL of aqueous HAuCl_4_ solution (25 mM) was added into 96 mL of boiling water with noticeable bubbling under stirring, followed by further boiling for at least 10 min. Subsequently, 3 mL of aqueous solution of sodium citrate (1 wt%) were rapidly injected into the boiling HAuCl_4_ solution under vigorous stirring. The color of the reaction solution changed from yellow to bluish-gray and then to ruby-red during the reaction process. The reaction solution was further refluxed for 30 min under stirring to warrant the formation of monodisperse, quasi-spherical AuNPs with a size of 15 nm. The reaction solution was cooled to room temperature for further use.

#### 3.4.2. Detection of E2 by Colorimetric Method Based on AuNPs

The presence of negatively charged citrate groups on the surface of AuNPs results in strong electrostatic repulsion between the particles, causing them to disperse uniformly in the solution. The uniform dispersion imparts a red color to the solution. Nevertheless, the addition of sodium chloride solution to the dispersed AuNPs solution can lead to the destabilization of the nanogold system. This destabilization causes AuNPs to aggregate, resulting in a change of solution color from red to blue. Single-stranded nucleic acid aptamers can effectively prevent the aggregation of AuNPs. These aptamers work by exposing the base in the AuNPs solution, allowing them to be adsorbed on the surface of the negatively charged AuNPs through the van der Waals force. The adsorption of aptamers on the AuNPs surface has a protective effect that helps the particles to remain dispersed, thereby maintaining the red color of the solution. The presence of E2 in the system can disrupt the protective effect of aptamers on the nanogold particles. Specifically, aptamers preferentially bind to E2, resulting in the disappearance of their protective effect on the AuNPs. Consequently, the AuNPs start to agglomerate under the influence of salt ions, causing the solution to change color from red to blue.

### 3.5. Fabrication of Paper-Based Aptasensor for Electrochemical Detection of E2

#### 3.5.1. Preparation of the EAB Sensor

The HEV1 aptamer was used to build a label-free electrochemical aptamer sensor for E2 detection. The paper-based sensor substrate and the three electrodes were designed using Adobe Illustrator CC 2018, as shown in Figure S1. The white area of the chip is a hydrophilic area, and the blue area is a hydrophobic area. The paper base aptasensor is made up of three layers. The two electrodes are printed with carbon ink and silver/silver chloride ink. Moreover, the third layer is working electrodes printed with carbon ink. The three-layer paper base is combined with double-sided tape to form a paper chip. When the hydrophilic area of the paper chip is full of samples, the device can accomplish the detection. Furthermore, the working electrode was modified with rGO/THI/AuNPs nanomaterials. HEV1 aptamer was immobilized on the electrode surface by the combination of AuNPs through the Au-S bond. When E2 binds to the aptamer, a conformational change occurs in the aptamer, resulting in a change of the steric hindrance on the electrode surface, causing a current signal change. This method not only eliminates the influence of nucleotide modification on the performance of the aptamer but also simplifies the experimental steps.

#### 3.5.2. Synthesis of rGO/THI/AuNP Nanocomposite

The rGO/Thi/AuNPs nanocomposites were synthesized at room temperature through the following process. First, 2.0 mg of rGO is dispersed into 2.0 mL of ultrapure water and sonicated for half an hour to disperse it evenly. Then, 2.0 mL of thionine solution (2.0 mg/mL) was added to this dispersion. The above mixture was vigorously stirred at 25 °C for 24 h. As a result of the π-π stacking interaction between thionine and graphene, rGO/THI nanocomposites were obtained. The as-prepared solution was mixed with AuNPs (a diameter of 15 nm) solution in a ratio of 1:5 and was stirred at 25 °C for 12 h prior to use.

#### 3.5.3. Fabrication of Aptasensor

First, 10.0 μL of the synthesized rGO/THI/AuNP nanocomposite was coated on the surface of the working electrode and dried in an oven at 50 °C for 4 h. Subsequently, 10 μL of the HEV1 aptamer was added to the surface of the working electrode and was immobilized on AuNP through Au-S bonds. Finally, 10 μL of MCH (1%) was dropped on the electrode surface to block the remaining active sites.

#### 3.5.4. Estradiol Detection Based on Electrochemical Aptasensor

The performance of this aptamer sensor was evaluated by analyzing the standard solution of E2. The characterization of the device was studied via cyclic voltammetry (CV) and differential pulsed voltammetry (DPV). CV was conducted with a scan rate of 100 mV s^−1^, and DPV was conducted with a scan rate of 10 mV s^−1^, with a modulation amplitude of 0.05 V, a modulation time of 0.025 s, and an interval time of 0.5 s.

## 4. Conclusions

In this study, to overcome the high synthesis cost and weak stability in the blood of long-chain aptamers, the M70 was structurally modified. And a shortened aptamer 22-mer HEV1 from the 76-mer aptamer was successfully identified. Firstly, affinity and KD values between HEV1 and E2 were determined. Then, the M70 and HEV1 were adopted to fabricate AuNPs-based colorimetric sensors and electrochemical sensors. A comparison between HEV1 and the 76-mer aptamer indicated that HEV1 behaves similarly to the parent aptamer regarding binding affinity and selectivity, from which the binding motif in the 76-mer aptamer was identified. There are three major innovations in this work: (1) A novel short aptamer of 17β-estradiol was developed and its binding ability was determined. (2) Colorimetric sensors and electrochemical sensors based on the representative aptamer and the novel aptamer were successfully fabricated. Both of these sensors show good performances. (3) In the stem–loop structure of HEV1, the sequences of the stem and the two ends can be further modified for many other aptasensor applications based on different mechanisms, such as electrochemical and fluorescent techniques for E2 detection.

## Figures and Tables

**Table 1:** Assay results for the clinical serum samples using a commercial instrument (reference concentration) and the proposed electrochemical sensor.

	Reference Concentration (pg/mL)	Electrochemical Sensor Measured Concentration (pg/mL)	Relative Error (%)
1	14.7	15.63	6.31
2	391.9	348.35	−11.11
3	523.5	430.87	−17.69
4	1202	1301.66	8.29
5	2642.3	2858.51	8.18

## Data Availability

The data presented in this study are available in article and Supplementary Materials.

## Supplementary Materials

The following supporting information can be downloaded at: https://www.mdpi.com/article/10.3390/molecules29020535/s1. Figure S1: Schematic diagram of sensors and modifications, as well as typical signal responses; Figure S2: Two dimensional structural simulation diagrams of (A) HEV1 and (B) HEV2; Figure S3: Schematic diagram of colorimetric method; Figure S4: (A) Transmission electron microscopy characterization of AuNPs; (B) Ultraviolet–visible light absorption spectrum of AuNPs; Figure S5: The calibration plots of peak current (Idpv) and E2 concentration (CE2) in clinical serum samples for the detection of E2 by (A) M70 aptasensor and (B) HEV1 aptasensor; Figure S6: (A) DPV response of three independent chips detecting 1 ng mL^−1^ E2; (B) Signals of the device to 1 ng/mL E2 and the mixed liquor of 1 ng/mL E2 with E3, E1, and PRO separately; Table S1: Comparation of KD value between kinds of aptamers with E2. Ref. [36] is cited in Supplementary Materials.
