# Supplementary material for: Design and Validation of a Short Novel Estradiol Aptamer and Exploration of Its Application in Sensor Technology
Source: Molecules. 2024 Jan 22;29(2):535. doi: 10.3390/molecules29020535 (PMC10819485; doi:10.3390/molecules29020535)
Supplement: Supplementary file 1 [file molecules-29-00535-s001.zip › molecules-2733344-supplementary.pdf]

Supporting Information

Design and Validation of a Short Novel Estradiol Aptamer and Exploration of Its Application in Sensor Technology

Hongyan Jin [1,*,†], Yan Cheng [1,†], Fanli Kong [2,3], He Huang [1], Zhenjun Yang [4], Xinyi Wang [2,3], Xinxia Cai [2,3], Jinping Luo [2,3] and Tao Ming [5,*]

[1] Obstetrics and Gynecology Department, Peking University First Hospital, Beijing 100034, China
[2] State Key Laboratory of Transducer Technology, Aerospace Information Research Institute, Chinese Academy of Sciences, Beijing 100190, China
[3] University of Chinese Academy of Sciences, Beijing 100049, China
[4] State Key Laboratory of Natural & Biomimetic Drugs, School of Pharmaceutical Sciences, Peking University, No. 38 Xueyuan Road, Haidian District, Beijing 100191, China
[5] Institute of Transplantation Medicine, School of Medicine, Nankai University, Tianjin 300190, China
* Correspondence: maggijhy@163.com (H.J.); mingtao@nankai.edu.cn (T.M.)
† These authors contributed equally to this work.

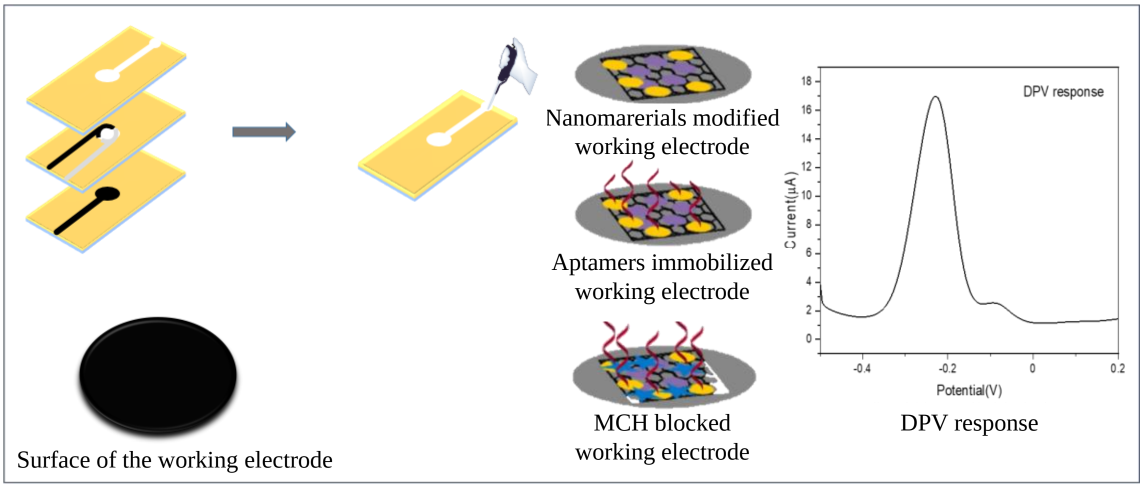

Figure S1. Schematic diagram of sensors and modifications, as well as typical signal responses.

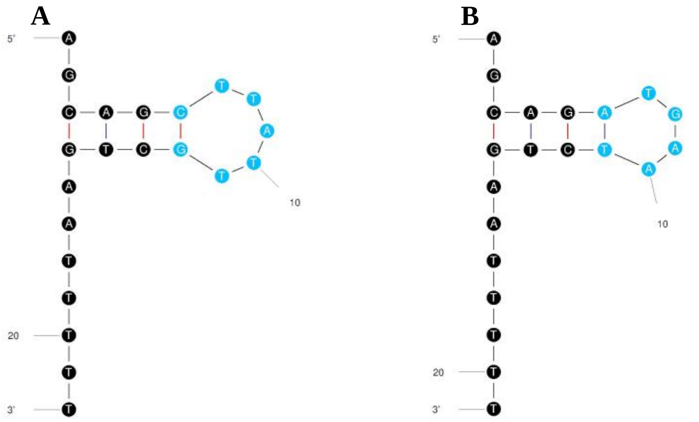

Figure S2. Two-dimensional structural simulation diagrams of (A) HEV1 and (B) HEV2.

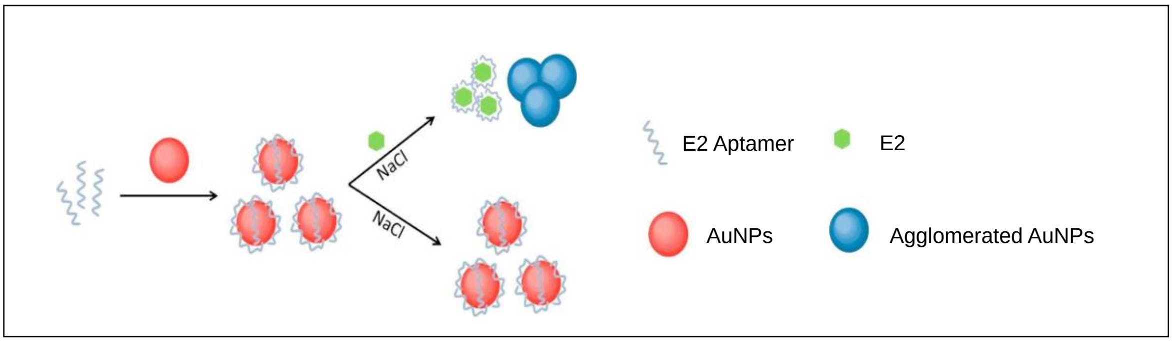

Figure S3. Schematic diagram of colorimetric method.

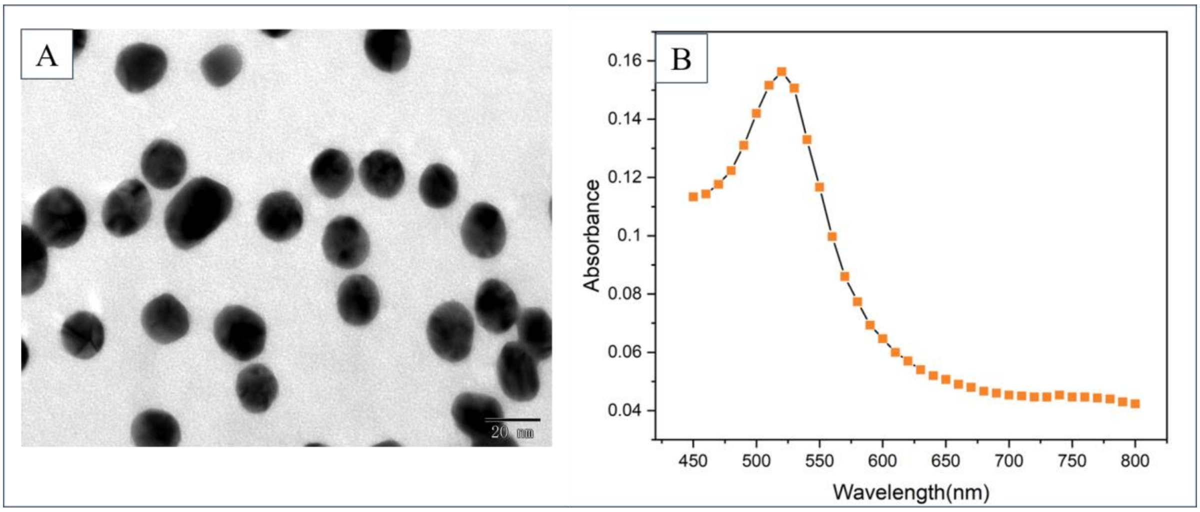

Figure S4. (A) Transmission electron microscopy characterization of AuNPs; (B) Ultraviolet-visible light absorption spectrum of AuNPs.

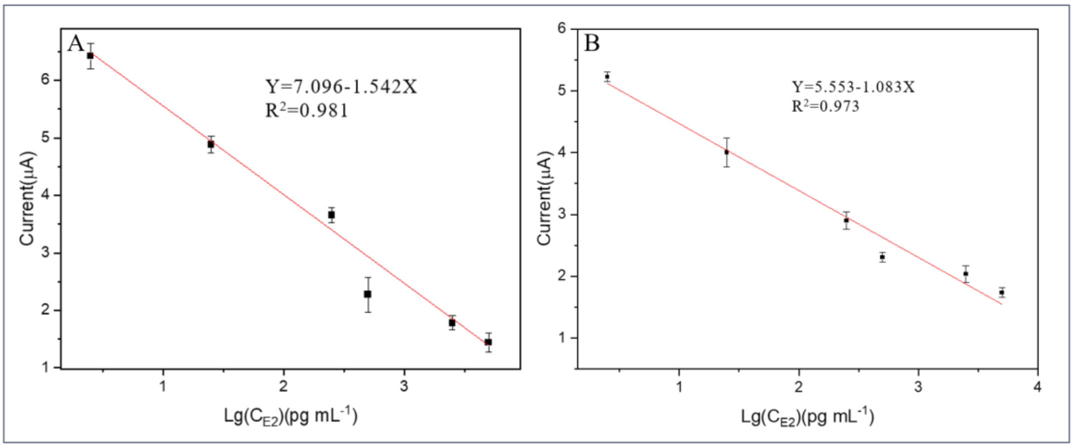

Figure S5. The calibration plots of peak current (I_{dpv}) and E2concentration (C_{E2}) in clinical serum samples for the detection of E2 by (A) M70 aptasensor and (B) HEV1 aptasensor.

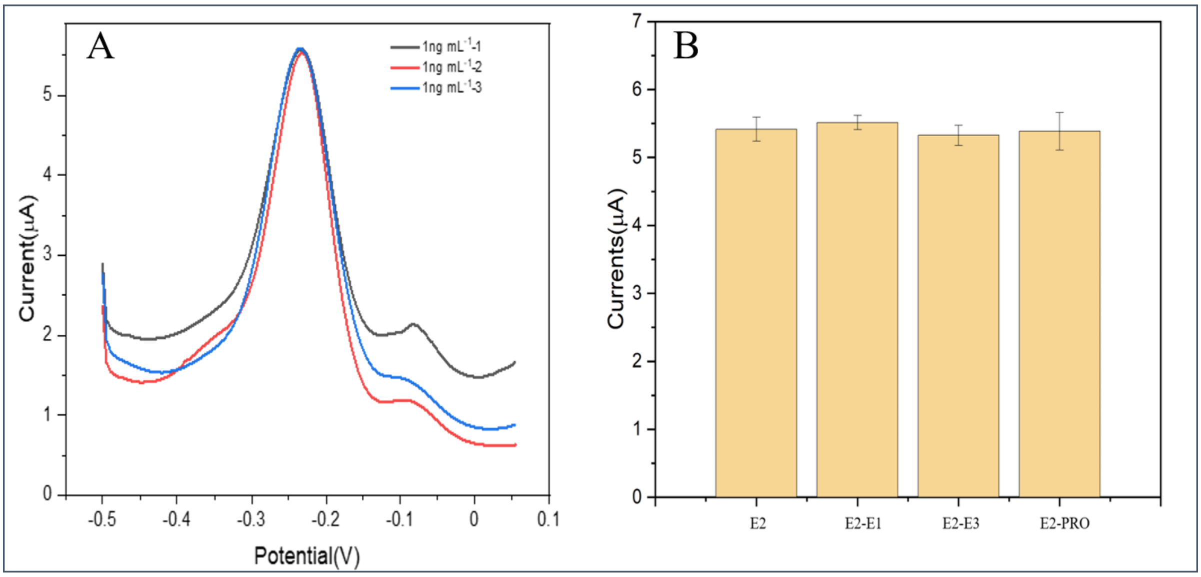

Figure S6. (A) DPV response of three independent chips detecting 1ng mL^{-1} E2; (B) Signals of the device to 1 ng/mL E2 and the mixed liquor of 1 ng/mL E2 with E3, E1, PRO separately;

Table S1. Comparation of KD value between kinds of aptamers with E2.

Aptamer	KD Value(nM)	Reference
Kim(76mer)	98±56	1
Alsager(75mer)	18±5	2
Alsager(35mer)	23±10	3
Alsager(22mer)	70±38	3
Akki	227±113	4
HEV1	92±66	This work

References

1. Kim, Y. S.; Jung, H. S.; Matsuura, T.; Lee, H. Y.; Kawai, T.; Gu, M. B., Electrochemical Detection of 17β-Estradiol Using DNA Aptamer Immobilized Gold Electrode Chip. *Biosensors and Bioelectronics* **2007,** *22* (11), 2525-2531.
2. Alsager, O. A.; Kumar, S.; Willmott, G. R.; McNatty, K. P.; Hodgkiss, J. M., Small Molecule Detection in Solution Via the Size Contraction Response of Aptamer Functionalized Nanoparticles. (1873-4235 (Electronic)).
3. Alsager, O. A.; Kumar, S.; Zhu, B.; Travas-Sejdic, J.; McNatty, K. P.; Hodgkiss, J. M., Ultrasensitive Colorimetric Detection of 17β-Estradiol: The Effect of Shortening DNA Aptamer Sequences. *Analytical Chemistry* **2015,** *87* (8), 4201-4209.
4. Akki, S. U.; Werth, C. J.; Silverman, S. K., Selective Aptamers for Detection of Estradiol and Ethynylestradiol in Natural Waters. *Environmental Science & Technology* **2015,** *49* (16), 9905-9913.
